# TR4 nuclear receptor enhances the cisplatin chemo-sensitivity *via* altering the ATF3 expression to better suppress HCC cell growth

**DOI:** 10.18632/oncotarget.8525

**Published:** 2016-04-01

**Authors:** Jiliang Shen, Hui Lin, Gonghui Li, Ren-An Jin, Liang Shi, Mingming Chen, Chawnshang Chang, Xiujun Cai

**Affiliations:** ^1^ Chawnshang Chang Liver Cancer Center, Department of General Surgery, Sir Run-Run Shaw Hospital, Zhejiang University, Hangzhou 310016, China; ^2^ George Whipple Laboratory for Cancer Research, Departments of Pathology and Urology and The Wilmot Cancer Center, University of Rochester Medical Center, Rochester, NY 14642, USA; ^3^ Sex Hormone Research Center, China Medical University/Hospital, Taichung 404, Taiwan

**Keywords:** TR4, hepatocellular carcinoma, chemotherapy, TR4 response element, ATF3

## Abstract

Early studies indicated that TR4 nuclear receptor (TR4) may play a key role to modulate the prostate cancer progression, its potential linkage to liver cancer progression, however, remains unclear. Here we found that higher TR4 expression in hepatocellular carcinoma (HCC) cells might enhance the efficacy of cisplatin chemotherapy to better suppress the HCC progression. Knocking down TR4 with TR4-siRNA in HCC Huh7 and Hep3B cells increased cisplatin chemotherapy resistance and overexpression of TR4 with TR4-cDNA in HCC LM3 and SNU387 cells increased cisplatin chemotherapy sensitivity. Mechanism dissection found that TR4 might function through altering the ATF3 expression at the transcriptional level to enhance the cisplatin chemotherapy sensitivity, and interrupting ATF3 expression *via* ATF3-siRNA reversed TR4-enhanced cisplatin chemotherapy sensitivity in HCC cells. The *in vivo* HCC mouse model using xenografted HCC LM3 cells also confirmed *in vitro* cell lines data showing TR4 enhanced the cisplatin chemotherapy sensitivity. Together, these results provided a new potential therapeutic approach *via* altering the TR4-ATF3 signals to increase the efficacy of cisplatin to better suppress the HCC progression.

## INTRODUCTION

Hepatocellular carcinoma (HCC) is the fifth most common cancer in the world [1] and ranks third among the most lethal cancers worldwide [2]. HCC is an aggressive cancer with a high mortality rate and many cases were diagnosed at a late stage when curative therapies are not easily to obtain [3]. Chemotherapy with several cytotoxic agents, such as cisplatin, doxorubicin and 5-florouracil, are widely used to treat advanced HCC, but chemo-resistance is often observed in the majority of the patients [4, 5]. Thus, development of better chemotherapy strategies is urgently needed.

The TR4 nuclear receptor 4 (TR4) belongs to the nuclear receptor superfamily and was first cloned from human prostate and testis cDNA libraries [6]. Early studies suggested that TR4 might play important roles to alter several key signaling pathways via interacting with selective nuclear receptors including thyroid receptor, androgen receptor, retinoic acid receptor/retinoid X receptor, and estrogen receptor [7–9]. TR4 could influence oxidative stress- and ionizing radiation-induced damage [10], and altered TR4 led to change the chemo-resistance of PCa stem/progenitor cells [11].

Here we investigated the potential TR4 potential roles in the HCC progression, especially its impacts on the cisplatin chemotherapy sensitivity. The results suggest that TR4 may function through altering the ATF3 expression to enhance the cisplatin chemotherapy sensitivity to suppress the growth of HCC cells.

## RESULTS

### TR4 expression is higher in HCC than surrounding normal liver cells in HCC patients

**Figure 1:**
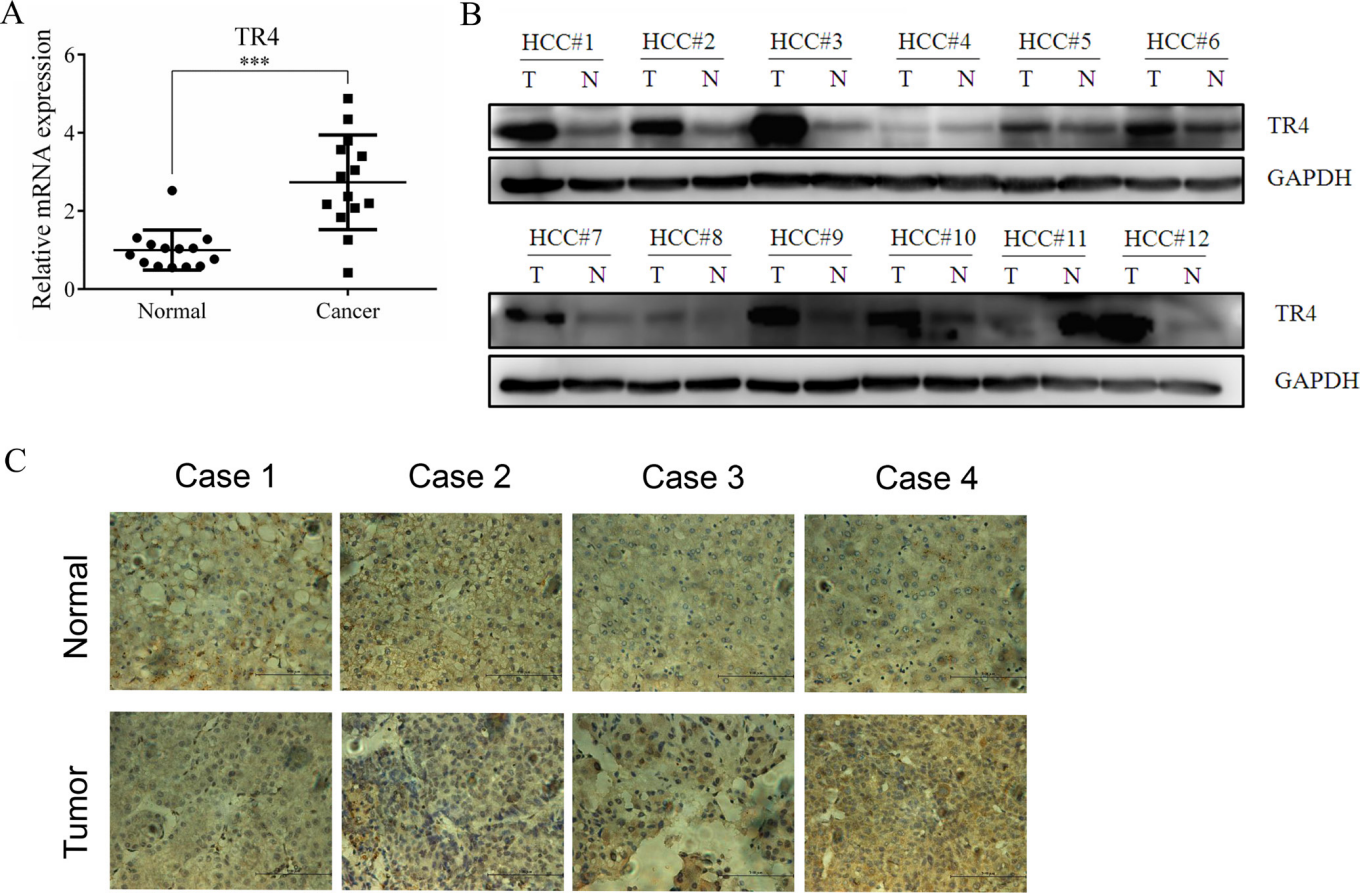
TR4 was up-regulated in tumor tissues among HCC patients (**A**) TR4 mRNA levels in 14 HCC patients. TR4 mRNA levels in tumor and surrounding non-tumor tissues were evaluated using real-time RT-PCR analysis as indicated, GAPDH showed stable expression in the conditions applied and was used as a reference gene. Mean threshold cycle (Ct) number of triplicate runs were used for data analysis. The relative expression of TR4 was calculated compared with the reference gene (GAPDH). (**B**) TR4 protein expression levels in tumor (T) and surrounding non-tumor (N) tissues were evaluated using Western blot analysis in another 12 patients as indicated. GAPDH served as a loading control. (**C**) TR4 staining in non-tumor lesions (upper panel), with less in tumor lesions (lower panel). All assays were performed in triplicate (***P < 0.001).

To study roles of TR4 in HCC progression, we first examined the TR4 expression in HCC patients liver samples, and results revealed higher TR4 mRNA expression in HCC as compared to surrounding normal liver cells in 14 HCC patients (Figure 1A). We also found higher TR4 protein expression in HCC in 10 of 12 patients analyzed using western blot analysis (Figure 1B) or IHC staining (Figure 1C).

Together, results from Figure 1A–1C reveal that TR4 expression at both mRNA and protein levels is higher in HCC than surrounding normal liver cells, suggesting TR4 expression may be linked to the HCC development.

### Higher expression of TR4 mRNA and protein in HCC cell lines correlate with greater cell chemo-sensitivity

**Figure 2:**
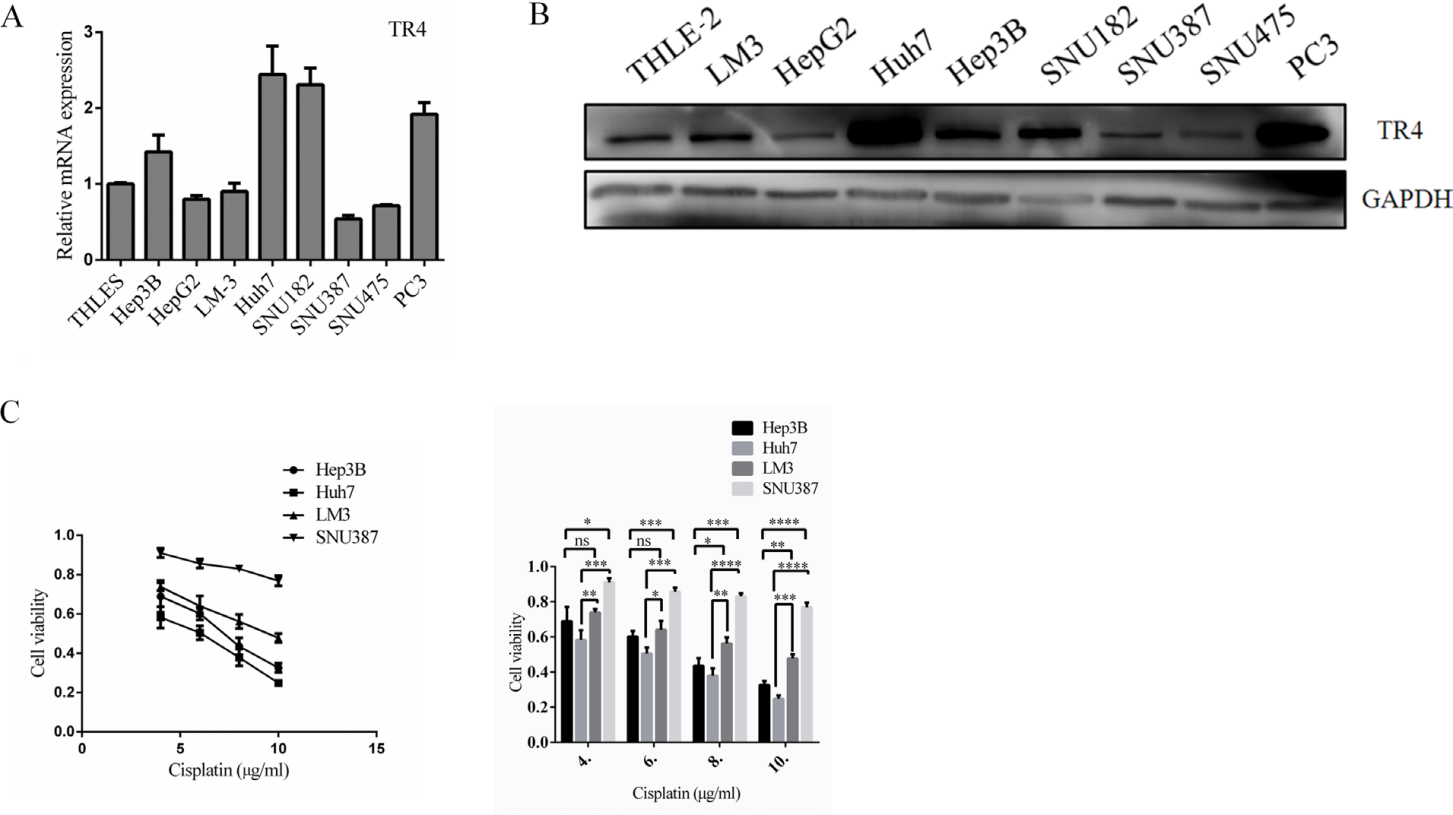
High TR4 mRNA and protein expression levels in HCC cell lines correlated with high Chemosensitity (**A**) TR4 mRNA levels in 7 HCC cell lines. The normal liver cell line THLE-2 and the positive control cell line PC3 were evaluated using real-time RT-PCR analysis as indicated, and data values were normalized to the mRNA level of THLE-2. (**B**) TR4 protein expression levels in each HCC cell line, normal liver cell line THLE-2, and the positive control cell line PC3 were evaluated using Western blot analysis as indicated. GAPDH served as a loading control. (**C**) Drug sensitivity test for cisplatin (CDDP) in Hep3B, Huh7, LM3, and SNU387 cells. Cells were treated with various indicated concentrations of cisplatin for 48 h, and cell viability upon drug treatment was analyzed by an MTS assay. Quantitation is shown at right. All assays were performed in triplicate (*P < 0.05, **P < 0.01, ***P < 0.001 ***P < 0.001, ns = not significant).

We first examined the TR4 expression in various HCC cell lines and found TR4 expression was higher in Hep3B and Huh7 cells and lower in LM3 and SNU387 cells (Figure 2A, 2B). We then studied differential expression of TR4 impacts on altering the cell viability upon chemotherapy. We found adding cisplatin, the current used chemotherapy drug to treat HCC [12], suppressed HCC cells using MTS assays (Figure 2B). Importantly, we found the cell viability was much higher in LM3 and SNU387 cells than in Huh7 and Hep3B cells (Figure 2C), suggesting higher TR4 expression in HCC cells may be able to increase cisplatin chemotherapy sensitivity to better suppress HCC cells.

### TR4 knockdown led to reduced chemo-sensitivity in Huh7 and Hep3B cells

**Figure 3:**
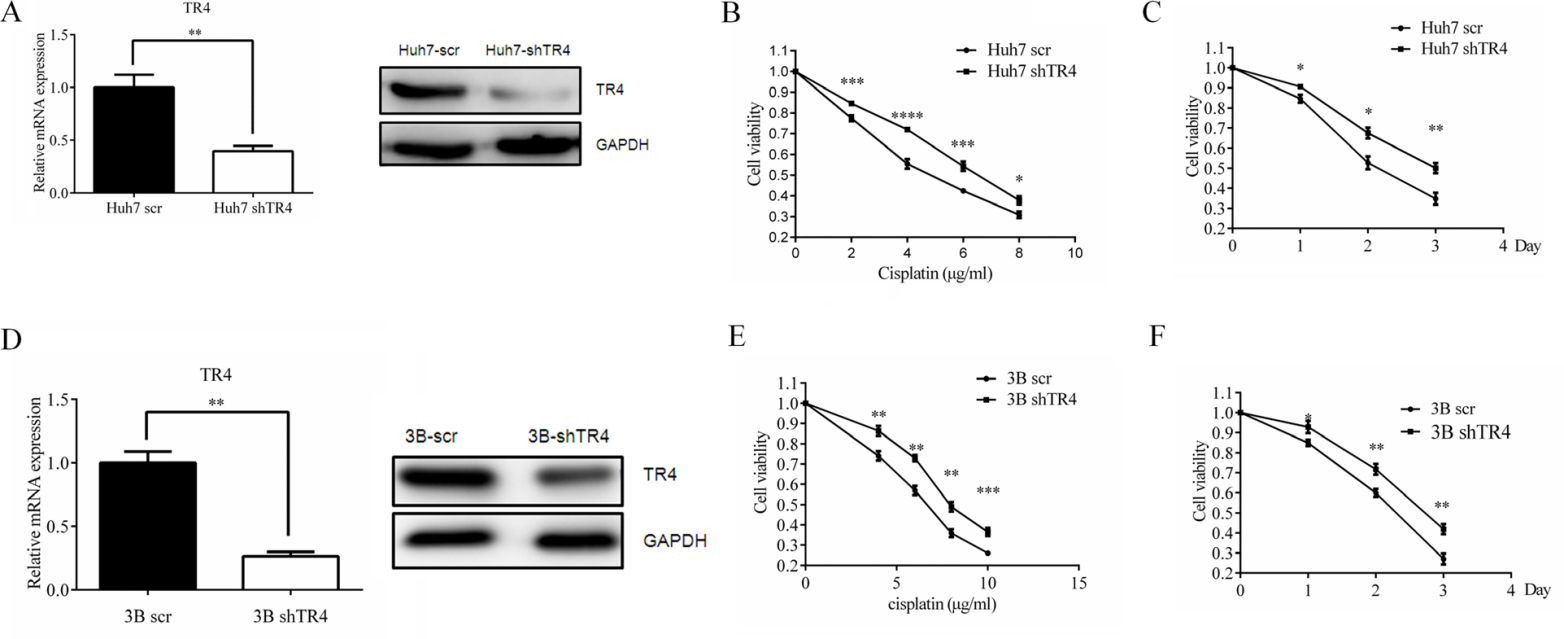
TR4 knockdown led to weakened chemosensitivity of Huh7 and Hep3B cells (**A**) qPCR and Western blot analysis results showing successful TR4 knockdown in Huh7 cells. Huh7 were infected with lentivirus carrying either sh-TR4 or scrambled (scr) control sequence, and TR4 mRNA and protein levels were analyzed by qPCR and Western blot analysis, respectively. GAPDH served as a control in analyses. (**B**, **C**) drug sensitivity test for cisplatin in Huh7-shTR4 and Huh7-scr cells. Cells were treated with various indicated concentrations of Line 5 should read drugs for 48 h (left panel) or treated with 4 μg/ml cisplatin (right panel) and analyzed every 24 h for 3 days, cell viability upon drug treatment was analyzed by an MTS assay. (**D**) qPCR and Western blot analysis results showing successful TR4 knockdown in Hep3B cells were infected as in (A) and TR4 mRNA and protein levels were analyzed by qPCR and Western blot analysis, respectively. GAPDH served as a control in analyses. (**E**, **F**) drug sensitivity test for cisplatin in Hep3B-shTR4 and Hep3B-scr cells. Cells were treated as in (B–C) for 48 h or treated with 6 μg/ml cisplatin and analyzed as in (B–C). All assays were performed in triplicate (*P < 0.05, **P < 0.01, ***P < 0.001).

To further confirm the above conclusion, we first knocked-down TR4 expression *via* TR4-shRNA in Huh7 cells (Figure 3A, mRNA level and protein level), and then treated these cells with cisplatin and applied MTS assay to analyze the cytotoxicity of these cells. We found that Huh7 cells have less sensitivity to cisplatin treatment in the TR4 knocked-down (Huh7-shTR4) cells compared with the scrambled control (Huh7-scr) cells (Figure 3B, 3C). Similar results were obtained when we replaced Huh7-shTR4 cells with Hep3B-shTR4 cells (Figure 3D–3F). Similar results were obtained when we used another knocked-down TR4 plasimid (Supplementary Figure S1).

### TR4 over-expression led to enhanced chemo-sensitivity in LM3 and SNU387 cell

**Figure 4:**
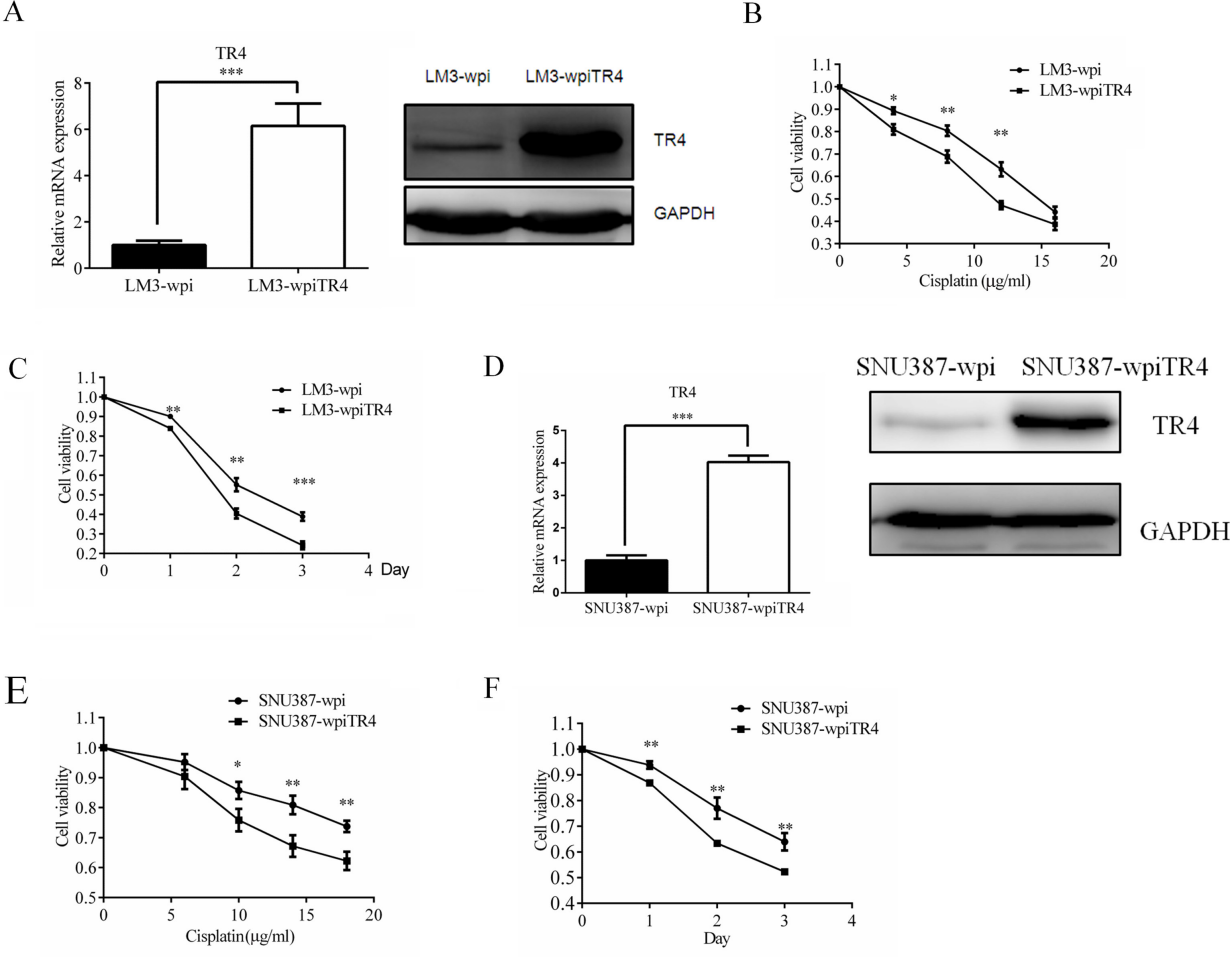
TR4 overexpression led to enhanced chemosensitivity of LM3 and SNU387 cells (**A**) qPCR and Western blot analysis results showing successful TR4 overexpression in LM3cells. LM3 were infected with lentivirus carrying either wpiTR4 or wpi control sequence, and TR4 mRNA and protein levels were analyzed by qPCR and Western blot analysis, respectively. GAPDH served as a control in analyses. (**B**, **C**) drug sensitivity test for cisplatin in LM3-wpiTR4 and LM3-wpi cells. Cells were treated with various indicated concentrations of cispaltin for 48 h or treated with 12 μg/ml cislatin and analyzed every 24 h for 3 days, cell viability upon drug treatment was analyzed by an MTS assay. (**D**) qPCR and Western blot analysis results showing successful TR4 overexpression in SNU387 cells. SNU387 were infected as in (A) and analyzed by qPCR and Western blot analysis. GAPDH served as a control in analyses. (**E**, **F**) drug sensitivity test for cisplatin in SNU387-wpiTR4 and SNU387-wpi cells. Cells were treated with various indicated concentrations of cisplatin for 48 h or treated with 18 μg/ml cisplatin and analyzed every 24 h for 3 days, cell viability upon drug treatment was analyzed by an MTS assay. All assays were performed in triplicate (*P < 0.05, **P < 0.01, ***P < 0.001).

We then applied an opposite approach to overexpress TR4 via adding TR4-cDNA in LM3 and SNU387 cells (Figure 4A, mRNA level and protein level), and treated these cells with cisplatin and used MTS assay to analyze the cytotoxicity of these cells. The results revealed that adding TR4 increased LM3 cell sensitivity to cisplatin treatment (Figure 4B, 4C). Similar results were obtained when we replaced LM3-wpi TR4 cells with SNU387-wpi TR4 cells (Figure 4D–4F).

### TR4 expression alters cell apoptosis of HCC cells treated with cisplatin

**Figure 5:**
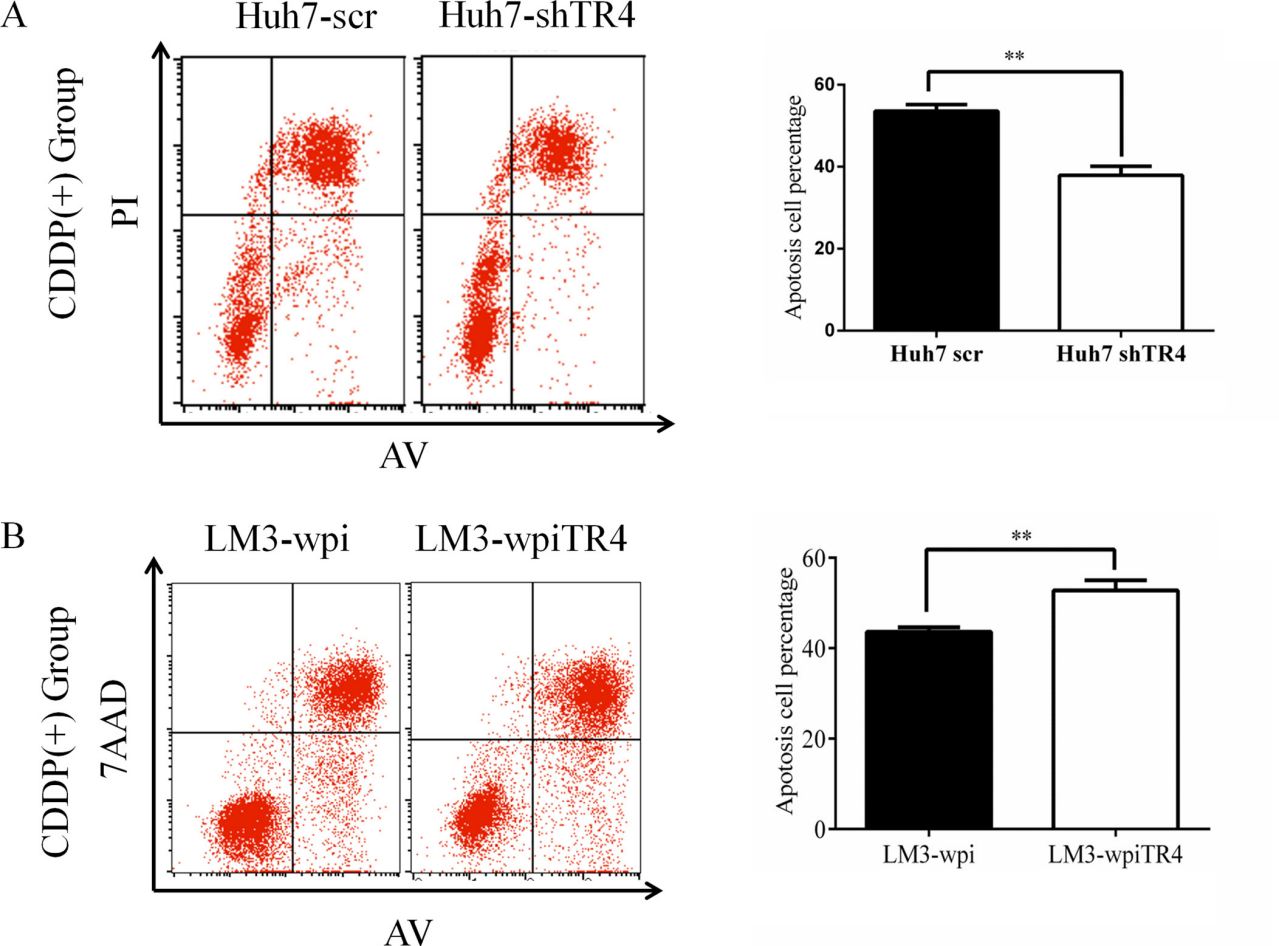
Higher TR4 expression resulting in a higher cell apoptosis in HCC cells treated with cisplatin (**A**) Huh7-scramble/shTR4 cells were treated with 6 μg/ml cisplatin for 48 h. Apoptosis was assessed as described in Materials. Quantification is shown on the right. Comparison among groups was performed using Student's test. (**B**) LM3-vector/TR4 cells were treated with 10 μg/ml cisplatin for 48 h. Flow cytometry (left) and comparison among groups (right) are shown as in (A). All assays were were performed in triplicate (*P < 0.05, **P < 0.01, ***P < 0.001).

We also performed apoptosis assays using Annexin V-FITC/PI double staining in both TR4 knocked-down cells and TR4 overexpressed cells. The results revealed that in TR4 knocked-down cells (Huh7-shTR4), cisplatin-induced apoptotic death is significantly less than scramble control (Huh7-scramble) (Figure 5A). In contrast, the apoptotic death was significantly more in TR4-overexpressed cells (LM3-wpiTR4) than vector control (LM3-wpi) (Figure 5B). These results demonstrate that TR4 can enhance the chemo-sensitivity of cisplatin possibly by promoting cell apoptosis.

### Mechanism dissection how TR4 alters the cisplatin chemo-sensitivity to HCC cells

Overwhelming evidence indicates that the *ATF3* gene can be induced rapidly by a variety of stress stimuli in different cell types possibly *via* altering cell apoptosis [13–21]. Germain et al. [22] also reported that cisplatin might suppress tumor growth *via* altering the *ATF3* expression and knocked-down *ATF3* might lead to attenuate the cisplatin-induced cytotoxicity in murine embryonic fibroblasts.

**Figure 6:**
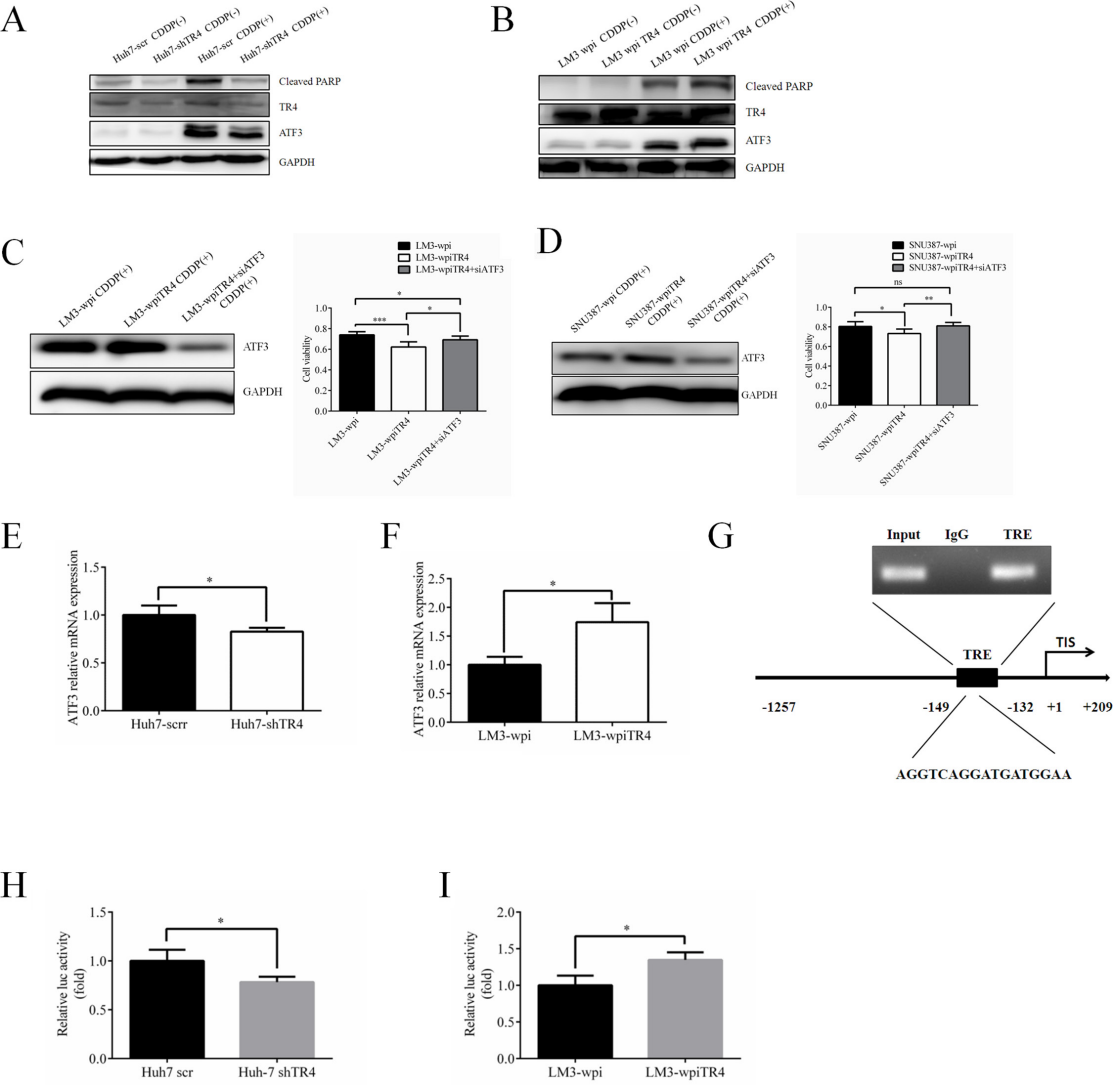
TR4 contributes to the chemosensitivity in HCC cells through up-regulation of ATF3 expression (**A**) Western blot analysis results show lower expression of ATF3 and cleaved PARP in TR4 knockdown Huh7 cells. Expression of TR4, ATF3, and cleaved PARP in Huh7-shTR4/Huh7-scr cells were shown. (**B**) Western blot analysis results show higher expression of ATF3 and cleaved PARP in TR4 overexpression HCC cells. Expression of TR4, ATF3, cleaved PARP in LM3-shTR4/LM3-scr cells were shown. (**C**, **D**) Effect of ATF3 interruption in neutralizing enhanced chemosensitity induced by TR4 overexpression in LM3 cells and SNU387 cells respectively. Western blot analysis of TR4 and ATF3 protein levels in LM3 cells, infected with either shATF3 or vector. Cells were infected by siATF3 and then treated with indicated concentration of cisplatin for 48 h. Cell viability was analyzed by MTS assays. (**E**, **F**) Q-PCR analysis results show lower expression of ATF3 in TR4 knocked down Huh7 cells and higher expression of ATF3 in TR4 overexpressed LM3 cells. (**G**) TR4 directly binds to the promoter of ATF3 through ChIP assay. Huh7 cells were cross-linked with formaldehyde (final 1%) for 15 min at room temperature and then subjected to ChIP assay using an anti-TR4 antibody or IgG (negative control) and the indicated primers. Reaction products were resolved by electrophoresis. The values presented are the means ± SD for each group (**H**) Huh7 cells were cultured and transiently transfected with ATF3-luciferase reporter without or with knocking down TR4 expression. (**I**) LM3 cells were cultured and transiently transfected with ATF3-luciferase reporter without or with overexpressing TR4 expression. (*P < 0.05, **P < 0.01).

We first found that *ATF3* expression was induced by cisplatin treatment and knocked-down TR4 significantly reduced its expression in the Huh7-shTR4 cells as compared with Huh7-scr cells (Figure 6A). Cell apoptosis using cleaved-PARP also found that cisplatin-induced ATF3 is higher in Huh7-scr (Figure 6A). As expected, overexpressing TR4 in LM3-wpiTR4 increased ATF3 expression as compared with LM3-wpi cells (Figure 6B).

We then applied neutralization/interruption approaches to confirm if TR4 is required to alter the ATF3 signaling to modulate the cisplatin chemo-sensitivity in HCC cells. As shown in (Figure 6C), we found interrupting *ATF3* expression in the LM3-wpiTR4 cells reversed the overexpressed TR4-enhanced cisplatin chemo-sensitivity in HCC LM3 cells, suggesting that TR4 might function through altering the ATF3 expression to enhance cisplatin chemo-sensitivity in HCC LM3 cells. Similar results were obtained when we replaced LM3 cells with SNU387 cells (Figure 6D).

Since the major biological function comes from transcriptionally regulating downstream genes, we assumed TR4 could directly bind to the promoter region of *ATF3* to influence its transcription. We first demonstrated that TR4 altered the *ATF3* expression at the transcriptional level in Huh7 cells and LM3 cells (Figure 6E, 6F). We analyzed the sequence of ATF3 promoter region seeking for potential TR4-response-element (TR4RE) based on a previous publication [23] and identified a putative TR4RE with the sequence of 5ʹ-AGGTCAGGATGATGGAA-3ʹ. We then applied the ChIP assay and found TR4 could bind to this TR4RE *in vivo* (Figure 6G). Finally, we constructed the reporter gene with ATF3 promoter linked to luciferase reporter and assayed its transcriptional activity after altering the TR4 expression in Huh7 cells and LM3 cells (Figure 6H, 6I). The results proved the transcription activation of ATF3 was induced by TR4 (Figure 6E–6I).

### TR4 alters the cisplatin chemo-sensitivity in HCC mouse model

**Figure 7:**
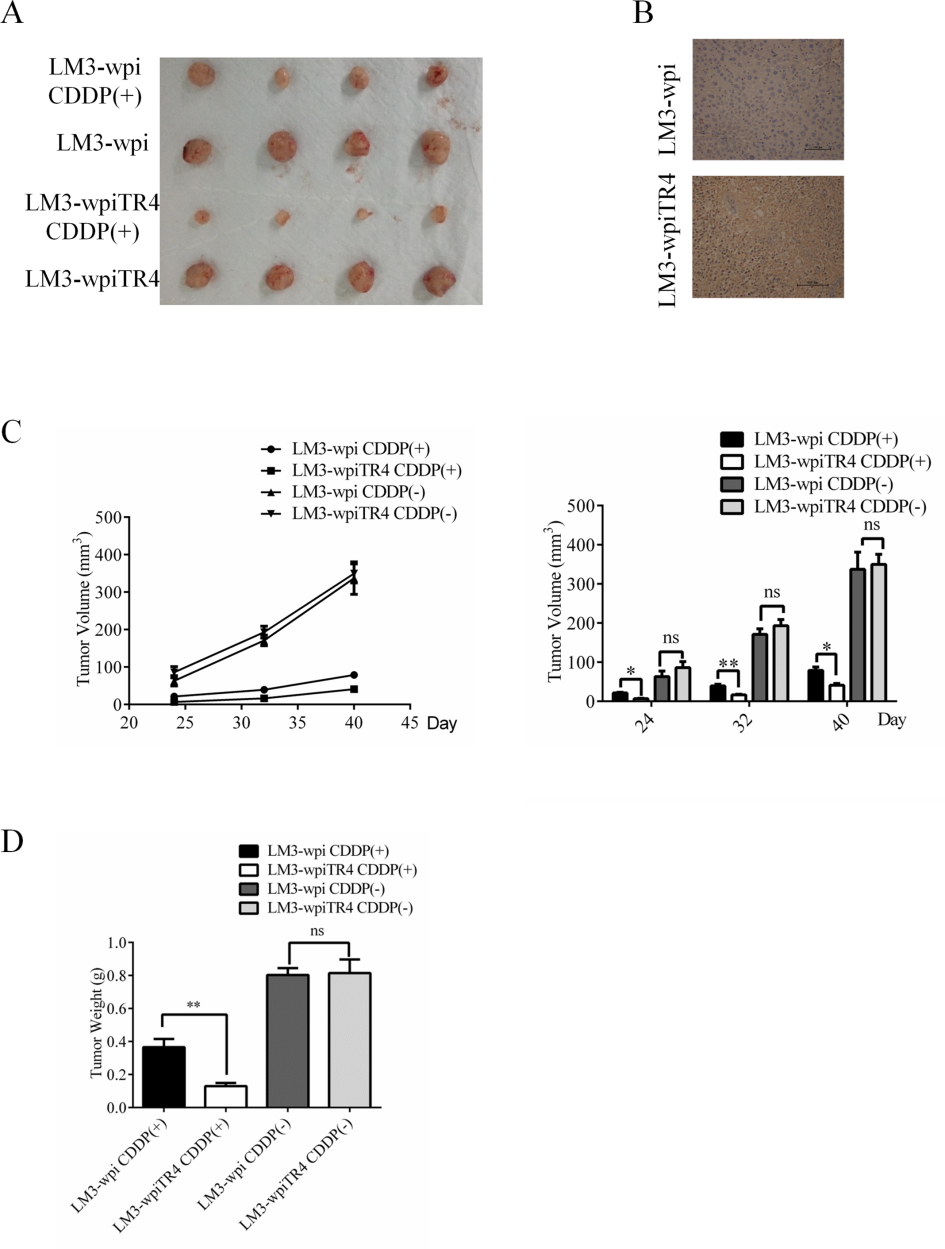
TR4 enhanced the effect of cisplatin in inhibiting the subcutaneous xenograft growth of Hepatocellular Carcinoma in nude mice Each mouse was injected subcutaneously with either LM3-wpi or LM3-wpiTR4 cells (2 × 10^6^ in 100 μl of medium) under the shoulder. Both groups were further divided into two subgroups (4 mice every group): control subgroup and treatment subgroup with cisplatin injection (1 mg/kg), every four days. The mice were euthanized after experiments, tumor tissues were excised and weighed. The original tumors (**A**), tumor tissues IHC of TR4 (**B**), tumor volumes (**C**), and tumor weights (**D**) were shown. The values presented are the means ± SD for each group (*P < 0.05, **P < 0.01, **P < 0.01, ns = not significant).

To confirm all above *in vitro* cell lines data *in vivo*, we generated the subcutaneous xenograft HCC mouse models by transplanting either LM3-wpi or LM3–wpiTR4 cells into nude mice. The nude mice were divided into two groups: LM3-wpi tumor group and LM3-wpiTR4 group. Both groups were further divided into two subgroups: control subgroup and treatment subgroup with 0.5 mg/kg body weight cisplatin (as known as CDDP) i.p injection. As shown in Figure 7A, significant inhibition of HCC growth was found in the two cisplatin treatment subgroups, and TR4 overexpression in the cisplatin treatment subgroup had better suppression of tumor growth.

IHC staining confirmed the different TR4 expression in the two groups (Figure 7B), and the LM3-wpiTR4 subgroup with cisplatin injection clearly had better suppression of the LM3 tumors (Figure 7C–7D).

## DISCUSSION

The efficacy of chemotherapy drugs varies greatly among individuals due to the heterogeneity of cancer biology, and many attempts have been developed to improve their chemo-sensitivity to better suppress the tumor growth [24–27]. For the HCC, it was reported that RYBP might be able to alter the HCC conventional chemotherapy *via* inducing HCC cells apoptosis [28]. Targeting NF-kappaB/miRNA-21/PTEN signaling pathway has also been suggested to alter the chemo-resistance [29].

Early studies indicated that TR4 might play cytoprotective roles [8, 10, 30–33] that could function through altering the cell survival signals including the TR4-Oct4-IL1Ra axis [11]. Here we found a reverse cytotoxic phenotype in HCC cells that might function through promoting cisplatin-mediated apoptosis, suggesting that the roles of TR4 in mediation of the tumor progression and chemotherapy efficacy is complex, and may depend on cell context or cytotoxicity inducers.

cisplatin has been used in the chemotherapy to suppress the HCC progression [34, 35]. Early studies indicated Cisplatin's anticancer activity was associated with its DNA damaging effects and induction of cancer cell apoptosis [36, 37], that might involve altering various signal transduction pathways, including activating ATF3 signaling pathways [22, 38]. Here we found that TR4 may alter the cisplatin chemotherapy efficacy through activating ATF3. By activating ATF3, TR4 promoted HCC cell apoptosis under chemo-treatment. Using luciferase assay and ChIP assay, we demonstrated that TR4 might function as a transcription factor via binding to the ATF3-TR4RE sequence that is located on its 5′ promoter region to regulate ATF3 expression in liver cells.

TR4 was first isolated as an orphan nuclear receptor without an identified ligand [6]. However, Xie et al. [23] found PPAR ligands/activators such as the PUFA metabolites, 15-HETE and 13-HODE, could trans-activate TR4, and some TZDs could also trans-activate TR4 to modulate its down-stream target CD36 activity during foam cell formation/atherosclerosis [39].

Interestingly, like other nuclear receptors that could also be trans-activated *via* phosphorylation, acetylation, or sumoylation [40], TR4 could also be modulated by the metformin that functions through activation of AMPK to phosphorylate TR4 [41]. Furthermore, androgen receptor co-activator ARA55 could also suppress TR4 transactivation by increasing acetylation in the DNA-binding domain of TR4 [42].

Here we found that TR4 may alter the cisplatin chemotherapy efficacy through activating ATF3. Future research using these potential TR4 ligands/activators or any potential upstream signals to increase TR4 function to increase cisplatin chemotherapy efficacy may help us to better suppress the HCC progression.

## MATERIALS AND METHODS

### Cell lines

The human HCC cell lines Hep3B, SNU182, SNU387, SNU475, and normal hepatic cell line THLE-2 were obtained from American Type Culture Collection (ATCC, Beijing, China) and Huh7, HepG2, and LM3 cell lines were obtained from the Type Culture Collection of the Chinese Academy of Sciences (Shanghai, China). LM3 cells were cultured in DMEM medium (Gibco, Shanghai, China) with 10% fetal bovine serum (FBS) (Gibco). Hep3B and HepG2 cells were cultured in MEM medium (Gibco, Shanghai, China) with 10% FBS. SNU182, SNU387, SNU475 and THLE-2 were cultured in 1640 medium (Gibco, Shanghai, China) with 10% FBS. The cells were maintained at 37°C in 5% CO2.

### Plasmids construction and cell infection

TR4-small hairpin RNA (shRNA) was cloned into pLKO.1 plasmid and TR4 gene was cloned in pWPI plasmid. Either scramble/vector control (pLKO.1-scramble/pWPI-vector) or shTR4/TR4 (pLKO.1-shTR4/pWPI-TR4) plasmids were transfected into 293T cells with a mixture of pKLO.1/pWPI, PAX2 (virus packaging plasmid) and pMD2G (envelope plasmid) (4:3:2 ratio) using Lipofectamine 2000 (Invitrogen). Lentiviral supernants were then collected to infect HCC cells. After viral infection, the media was replaced with normal culture media. The stable cells were selected and confirmed by quantative real-time PCR (qPCR) and western blot and then named as HCC-scramble/HCC-shTR4 and HCC-wpi/HCC-wpiTR4.

### Quantitative RT-PCR

Quantitative PCR was carried out using Taqman Gene Expression Assays (Applied Biosystem) or SYBP Green PCR amplification kit (Applied Biosystem). The primers of TR4 and glyceraldehyde 3-phosphate dehydrogenase (GAPDH) were designed by PrimerPremier 5.0 and synthetized by Biosune Biological Technology. The sequences of TR4 primers are: forward: 5ʹ-GGCTCTGAACCTGCCTCTG-3ʹ, reverse: 5ʹ-AGGATGAACTGCTGTTTGGG-3ʹ. The sequences of GAPDH primers are: forward 5ʹ-GGAGTCAACGGATTTGGT-3ʹ, reverse: 5ʹ-GTGATGGGATTTCCATTGAT-3ʹ. qPCR reaction condition: Step 1: 95°C, 2 min; Step 2: 95°C, 30 sec; 60°C 30 sec; 68°C, 1 min; 40 cycles; Step 3: 72°C, 10 min. The results were analyzed by delta–delta Ct method.

### Western blotting

Cells were harvested and washed twice with cold PBS, then resuspended and lysed in RIPA buffer (1% NP-40, 0.5% sodium deoxycholate, 0.1% SDS, 10 ng/ml PMSF, 0.03% aprotinin, 1 μM sodium orthovanadate) at 4°C for 30 min. Lysates were centrifuged for 10 min at 14,000 × g and supernatants were stored at −80°C as whole cell extracts. Total protein concentrations were determined by Bradford assay. Proteins were separated on 12% SDS-PAGE gels and transferred to polyvinylidene difluoride membranes. Membranes were blocked with 5% BSA and incubated with the indicated primary antibodies. Corresponding horseradish peroxidase-conjugated secondary antibodies were used against each primary antibody. Proteins were detected using the chemiluminescent detection reagents.

### Immunohistochemical staining (IHC)

We collected 90 tumor and normal samples from HCC patients at Sir Run-Run Shaw Hospital. IHC was then performed to evaluate TR4 expression in these samples. IHC was also performed in subcutaneous tumors of node mice to evaluate TR4 expression.

### *In vitro* cytotoxicity assay

Stable transfected cells (5 × 10^3^) were seeded on a 96-well plate with 3 replicate wells and allowed to incubate for 48 hr with the treatment of various concentrations of cisplatin or incubated for 72 hr with the indicated concentration of cisplatin and assessed every 24 hr. After incubation, cell viability was assessed utilizing the tetrazolium-based MTS colorimetric assay (CellTiter 96 cell proliferation assay kit; Promega, Madison, WI, USA) according to the manufacturer's instructions. All experiments were performed at least in triplicate on three separate occasions. Dose-response curves were plotted.

### Apoptosis assay

Cell apoptosis was evaluated by flow cytometry (FCM) assay. Briefly, Huh7-scr and Huh7-shTR4 cells were harvested and washed twice with PBS, stained with Annexin VFITC and propidium iodide (PI) in the binding buffer, and detected by FACS Calibur FCM (BD, CA, USA) after 15 min incubation at room temperature in the dark. LM3-wpi and LM3-wpiTR4 cells were harvested and washed twice with PBS, stained with Annexin V and 7-AAD in the binding buffer. The total apoptotic cells were quantified.

### Subcutaneous xenograft model

Animal studies were conducted using Female 5 week-old nude mice. Subcutaneous implantation was performed as previously described (23) where mice were injected subcutaneously with LM3-wpi or LM3-wpiTR4 cells. After a week, mice were randomized into two groups: cisplatin solution at the dose of 0.5 mg/kg in treatment group every four days nine times and DMSO control group in the same manner. The two perpendicular diameters (W and L) of tumors were recorded. The tumor volume (V) was calculated according to the formula: V = (W^2^ × L)/2. The mice were euthanized after experiments, and tumor tissues excised and weighed. All animal experiments were performed humanely in compliance with guidelines reviewed by the Animal Ethics Committee of the Biological Resource Centre of the Agency for Science, Technology and Research at the Sir Run-Run Shaw Hospital.

### Statistical analysis

All results are expressed as mean ± standard deviation (SD). Statistical analysis of the difference between treated and untreated groups was performed with Student's *t*-test. Values of *P* < 0.05 were considered as significant differences.

## SUPPLEMENTARY MATERIALS FIGURE


