# Supplementary material for: TR4 nuclear receptor enhances the cisplatin chemo-sensitivity via altering the ATF3 expression to better suppress HCC cell growth
Source: Oncotarget. 2016 Apr 1;7(22):32088–99. doi: 10.18632/oncotarget.8525 (PMC5077999; doi:10.18632/oncotarget.8525)
Supplement: Supplementary file 1 [file oncotarget-07-32088-s001.pdf]

TR4 nuclear receptor enhances the cisplatin chemo-sensitivity via altering the ATF3 expression to better suppress HCC cell growth

Supplementary Materials

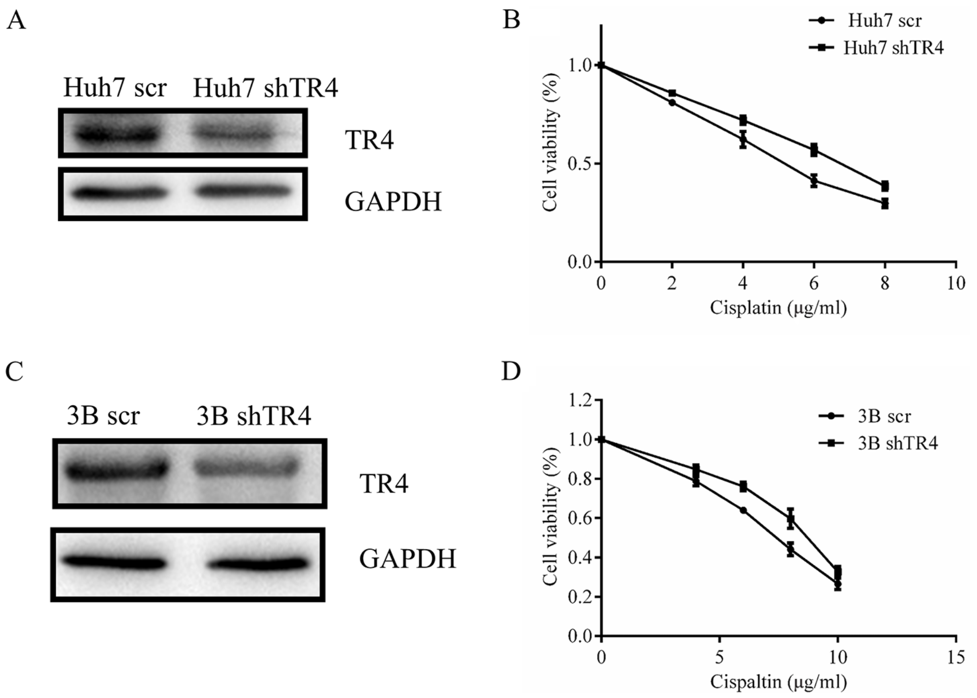

Supplementary Figure S1: TR4 knock down led to weakened chemosensitivity of Huh7and Hep3B cells using another knock down plasmid. (**A**, **C**) Western blot analysis results showing successful TR4 knock down in Huh7 and Hep3B cells. (**B**) Drug sensitivity test for cisplatin in Huh7-shTR4 and Huh7-scr cells. Cells were treated with various indicated concentrations of drugs for 48 h. (**D**) Drug sensitivity test for cisplatin in Hep3B-shTR4 and Hep3B-scr cells. Cells were treated with various indicated concentrations of drugs for 48 h. Cell viability upon drug treatment was analyzed by an MTS assay. All assays were performed in triplicate.
